# Finding the Best Way to Deliver Online Educational Content in Low-Resource Settings: Qualitative Survey Study

**DOI:** 10.2196/16946

**Published:** 2020-05-26

**Authors:** Lucy Kynge

**Affiliations:** 1 Interburns Swansea University Swansea United Kingdom; 2 The Centre for Global Burn Injury, Policy and Research Swansea University Swansea United Kingdom

**Keywords:** online education, digital content, health care, burns, low-resource settings

## Abstract

**Background:**

The reach of internet and mobile phone coverage has grown rapidly in low- and middle-income countries (LMICs). The potential for sharing knowledge with health care workers in low-resource settings to improve working practice is real, but barriers exist that limit access to online information. Burns affect more than 11 million people each year, but health care workers in low-resource settings receive little or no training in treating burn patients. Interburns' training programs are tailor-made to improve the quality of burn care in Asia, Africa, and the Middle East; the challenge is to understand the best way of delivering these resources digitally toward improved treatment and care of burn patients.

**Objective:**

The aim of the study, funded by the National Institute for Health Research (NIHR), was to understand issues and barriers that affect health care worker access to online learning in low-resource settings in order to broaden access to Interburns' training materials and improve burn-patient care.

**Methods:**

A total of 546 participants of Interburns' Essential Burn Care (EBC) course held in Bangladesh, Nepal, Ethiopia, and the West Bank, the occupied Palestinian Territories, between January 2016 and June 2018 were sent an online survey. EBC participants represent the wide range of health care professionals involved with the burn-injured patient. A literature review was carried out as well as research into online platforms.

**Results:**

A total of 207 of 546 (37.9%) participants of the EBC course did not provide an email address. Of the 339 email addresses provided, 81 (23.9%) “bounced” back. Surgeons and doctors were more likely to provide an email address than nurses, intern doctors, or auxiliary health care workers. A total of 258 participants received the survey and 70 responded, giving a response rate of 27.1%. Poor internet connection, lack of time, and limited access to computers were the main reasons for not engaging with online learning, along with lack of relevant materials. Computers were seen as more useful for *holding information*, while mobile phones were better for communicating and *sharing knowledge*. Health care workers in LMICs use mobile phones professionally on a daily basis. A total of 80% (56/70) felt that educational content on burns should be available through mobile apps.

**Conclusions:**

Health care workers in low-resource settings face a variety of barriers to accessing educational content online. The reliance on email for sign-up to learning management systems is a significant barrier. Materials need to be relevant, localized, and easy to consume offline if necessary, to avoid costs of mobile phone data. Smartphones are increasingly used professionally every day for communication and searching for information, pointing toward the need for tailored educational content to be more available through mobile- and web-based apps.

## Introduction

The reach and proliferation of the internet and mobile phones has expanded rapidly across low- and middle-income countries (LMICs), enabling broader access to educational opportunities and an attitude shift in how content can be successfully delivered. However, reaching specific audiences such as health care workers in low-resource settings to influence working practice is a multilayered challenge. Materials need to be contextually appropriate, interesting, and formatted to engage the user on their chosen device, but they must also be easy to access with minimal barriers.

Since 2012 when Massive Open Online Courses (MOOCs) were launched, there has been huge growth in online educational content and providers, but course completion rates for *industrial* or *mass media*-type e-learning models have been low, and dropout rates high, particularly in developing countries. Reasons range from socioeconomic, cultural, and administrative factors to contextual barriers, such as content that is inappropriate, or different pedagogical approaches. A study in 2018 states that while “the primary factors of adoption are perceived usefulness and perceived ease of use...there are several other factors which work along with these two factors to explain technology adoption” [1].

Health care workers in low-resource settings face particular challenges and often feel professionally isolated. There is little time, incentive, or, in many cases, connectivity, to go online to study, which means that materials need to be especially meaningful and accessible if they are to impact working practice. In the case of LMICs, the *small-scale* model is encouraged. Professor Deidre Carabine of the Virtual University of Uganda has coined the term “global knowledge for local action,” which encourages the contextualization and local creation of content in which quality and adaptability of educational programs are the key to success: “A major challenge...is to make online learning first-class, that’s: relevant, interesting, engaging, and more exciting than the traditional classroom...” [2].

Understanding how health care workers in resource-poor settings can access online content has been the focus of a study by Interburns, a UK charity working closely with the Centre for Global Burn Injury and Policy Research (CGBIPR) at Swansea University and funded by the National Institute for Health Research (NIHR). Interburns works with partners in Asia, Africa, and the Middle East to develop and deliver contextualized face-to-face training programs to tackle the global burden of burn injuries through capacity development and quality improvement. Burns affect more than 11 million people annually, with 95% occurring in poor countries, and the majority of those affected are children. Interburns’ tailored training programs focus on the reality of burn care services on the ground in low-resource settings and emphasize the need for *knowledge turned into action*.

The challenge is how to expand the reach of Interburns’ face-to-face training materials in an easily acceptable online format. This research focused on understanding barriers that health care workers involved with burn-injured patients faced when accessing online content in Bangladesh, Ethiopia, Nepal, and the West Bank, the occupied Palestinian Territories. The information gathered has been used to reformat the Essential Burn Care (EBC) Training Manual as an online resource and to inform a digital strategy for all of Interburns’ training resources.

## Methods

An online survey was carried out between July and September 2018 to understand the way in which health care workers in low-resource settings engage with digital content and to understand common barriers to access. Questions focused on previous experiences with online education, reasons why online education had been seen to be successful or not, attitudes and access issues around computers and mobile phones, and suggestions of ways in which information on burns could best be delivered.

A total of 546 participants of Interburns’ face-to-face course in EBC from Bangladesh, Nepal, Ethiopia, and the Palestinian West Bank between January 2016 and June 2018 were selected to participate in the survey. These health care workers represent the wide range of professions involved with the burn-injured patient; they are also the intended target of the digital version of the EBC Training Manual. Consent was given at the time of the training course and in the survey.

Following a review of survey results in December 2018 and a literature review, the EBC Training Manual was reformatted for an identified online platform and trialed with health care workers in 2019.

## Results

A total of 207 of the 546 participants (37.9%) of Interburns’ EBC training courses did not provide an email address when asked to leave contact details at the end of those courses. Out of 546 participants, 339 (62.1%) gave an email address, but 81 of those 339 emails (23.9%) with the survey link “bounced” back. This suggests that the email address given was incorrectly remembered or no longer in use. This is likely to show that a substantial number of health care workers in these settings do not regularly use email. Surgeons and doctors were more likely to give an email address than nurses, intern doctors, auxiliary health care workers, or medical officers. Out of 220 participants from Bangladesh, 118 (53.6%) did not give an email address; of these, 88 (74.6%) were nurses. Out of 281 health care workers from Nepal, 74 (26.3%) did not give an email address; 31 of these (41.8%) were nurses.

Learning management systems (LMSs) require an email address for sign-up and verification of individual users. If online educational content is held on an LMS, a large proportion of health care workers in these settings will be unable to sign up for a course unless they have an email address. This is an immediate, though hidden, barrier to access.

Of 258 participants who received the survey 70 responded, giving a response rate of 27.1%. Responses were balanced between male and female health care workers from Bangladesh (13/70, 19%), Nepal (42/70, 60%), Ethiopia (6/70, 9%), and the Palestinian West Bank (3/70, 4%). A total of 6 respondents (9%) selected *other*. See Table 1 for details of the professions represented by the survey.

A total of 74% (52/70) of respondents said they had used computers for online learning, but poor internet connection, lack of time, and limited access to computers were cited as reasons for not engaging well or regularly with online learning. With the rise in mobile phone ownership, better connectivity, and the increasing use of smartphones, participants expressed the view that computers were useful for *holding information*, while mobile phones were better for communicating and *sharing knowledge*, as expressed in the following quotes:

Because of the development of mobile phones, people rarely need access to use computers. In contrast, mobile phones nowadays are an essential accessory.

Medical student, Chittagong, Bangladesh

Information is safely stored in computer, and information of mobile phone is portable.

Nurse, Central Development Region, Nepal

I prefer mobile phone because we always carry mobile phone everywhere, so we can get information easily anywhere.

Nurse, Kirtipur, Nepal

This was backed up by the data, which showed that mobile phones are increasingly used professionally by health care workers in low-resource settings.

A total of 83% (58/70) of participants use mobile phones to find information for personal use at least two or three times each week, and 30% (21/70) also use their phones to look for professional information. A total of 80% (56/70) felt that mobile apps could be used to convey information and education on burns. This suggests that content created for mobile phones and apps would have a strong chance of uptake. However, 33% (23/70) of respondents worried about the high costs of data that can be incurred when accessing content. Representative quotes are given below:

Messenger systems are used by almost everyone that has a smartphone; short messages and information can effectively be transferred and accessed.

Auxiliary health care worker, Oromia Region, Ethiopia

It will be more helpful to use messenger systems for burn care. It will be quick and immediate and also recent.

Health care manager, Bangladesh

Cost-benefit ratio is not satisfactory.

Surgeon, Comilla, Bangladesh

It may cause financial problem.

Physiotherapist, Tigray Region, Ethiopia

[There is a cost] when using mobile data for seeing informative videos.

Nurse, Kathmandu, Nepal

**Table 1:** Participant professions.

Profession	Value (N=70), n (%)
Nurse	25 (36)
Doctor	19 (27)
Intern doctor	1 (1)
Therapist	6 (9)
Surgeon	14 (20)
Registrar	1 (1)
Auxiliary health care	1 (1)
Other	3 (4)
Total	70 (100)

## Discussion

### Principal Findings

The online survey showed that a significant number of health care workers in low-resource settings do not use email. This is a major barrier to accessing educational materials, which are hosted on LMSs that rely on email for sign-up and verification. The survey also found that mobile phones are increasingly used for professional purposes, although the high cost of data is a potential barrier for users.

Creating meaningful and contextualized educational content is the vital first step, and content needs to be formatted to be easily accessible in the online environment; Depover and Orivel stated, “The quality, relevance, and adaptability of educational programmes are becoming more important than ever” [3]. Lack of time, poor connectivity, irrelevant content, and limited access to computers are common reasons for low uptake of online courses, but each microsetting can throw up its own barriers in LMICs.

A study in Bangladesh applied a critical realist approach, in which *entity*, *agency*, and *causality* were used to explain the layers of context influencing online learning in the workplace. The study found that factors such as no desks, no internet connection, background noise, and no technical support hindered success. Other demotivating factors included low salary, too much work, lack of monitoring, and lack of support from family members [4]. Veletsianos stated, “...technology and certain practices associated with it are often expected to revolutionize the way individuals learn and teach. Yet scholars and practitioners alike are wise to maintain some skepticism about promises of transformation that ignore the environmental factors that surround innovations” [5].

In LMICs, there are a variety of barriers to accessing online content and reaching a specific population within a particular setting; in this case, the health care worker treating burn patients is a special challenge. Material must be formatted for the screen of choice and delivered with as few barriers as possible. Internet access and cost of data are high on the list of barriers. Mobile phone coverage is still far from uniform even within localities, though this is improving. In Bangladesh, the number of internet subscribers has risen year on year by approximately 10 million, reaching 83 million in 2018. Penetration by mobile broadband has seen similarly rapid growth. However, internet use in Nepal is low by international standards at 55% penetration in 2017, although the mobile phone market has grown quickly.

With greater connectivity, mobile devices can be used to communicate educational health messages, including at the community or rural level. Mobile phone penetration has risen considerably among community health care workers (CHWs) in low-resource settings, where multiple studies use smartphone apps to monitor and record patient details. A study into HIV data-gathering across five districts in Malawi found that mobile phone ownership was 100%, with smartphone ownership at 80% among decision makers and 50% among CHWs [6].

There have been experiments linking traditional LMSs, designed for computer-first online learning, with mobile messenger apps. A study in China piloted an online LMS linked to the WeChat messenger system as the training platform. Course completion rates rose from over 60% to 100%, and 63% of participating nurses wanted to receive *push* notifications through their phones for upcoming training courses [7].

Malhotra states, “Mobile phones are among the most coveted items among youth” [8], which increases the potential for greater acceptance of educational content delivered on a handset. Potential drawbacks, however, can be out-of-date devices, the potential for distraction, and the blurring of barriers between personal and professional use. However, benefits of using a mobile-first approach include the potential for greater access, more situated and contextualized learning, convenience, communication, and interaction. While learners will increasingly expect all online learning to work seamlessly on a mobile phone, there are challenges to scaling up approaches, not least of which is sustainable financing for the large-scale use of mobile phone technology in resource-limited settings [9].

This online survey showed a common but potentially hidden barrier, which is that sign-up processes for LMSs require email links and passwords to verify user information. Email is less used in many LMICs, meaning that registering for an online course is instantly challenging. Furthermore, where training is focused on the acquisition of knowledge-based technical skills, such as those required in burn treatment and care, online achievements may not reflect actual ability in the workplace [10]. Blended programs of regular face-to-face support are necessary to translate online learning into daily practice and will also encourage sustained use of digital materials.

### Conclusions

Technological improvements have expanded the ways in which health care workers in low-resource settings can access knowledge to improve working practice, but each local setting can throw up barriers. It is vital to understand the context within which the individual accesses digital information so that materials can be tailored to the setting and screen of choice. LMSs use email for sign-up and verification, and passwords for course access. This is a significant barrier for health care workers in low-resource settings who are not used to using email in their daily lives.

Health care workers in LMICs increasingly use mobile messaging professionally, and the majority of those surveyed felt that educational information on burns should be made available on both computers and mobile devices. Digital content needs to be contextually appropriate, formatted to be interesting, and accessible by computer, but with a *mobile-first* approach to developing content. Blended programs of face-to-face and online training will encourage greater use of online resources.

## Abbreviations

CGBIPR: Centre for Global Burn Injury and Policy Research
CHW: community health care worker
EBC: Essential Burn Care
LMIC: low- and middle-income country
LMS: learning management system
MOOC: Massive Open Online Course
NIHR: National Institute for Health Research
